# Effects of Salt Stress on Salt-Repellent and Salt-Secreting Characteristics of Two Apple Rootstocks

**DOI:** 10.3390/plants13071046

**Published:** 2024-04-08

**Authors:** De Zhang, Zhongxing Zhang, Yanxiu Wang

**Affiliations:** College of Horticulture, Gansu Agricultural University, Lanzhou 730070, China; m18394189590@163.com (D.Z.); zzx19872354@163.com (Z.Z.)

**Keywords:** apple stock, salt stress, paraffin section, salt repellent ability, ability to secrete salt

## Abstract

The effects of NaCl-induced salinity on biomass allocation, anatomical characteristics of leaves, ion accumulation, salt repellency, and salt secretion ability were investigated in two apple rootstock cultivars (*Malus halliana* ‘9-1-6’ and *Malus baccata*), which revealed the physiological adaptive mechanisms of *M. halliana* ‘9-1-6’ in response to salt stress factors. This experiment was conducted in a greenhouse using a nutrient solution pot. Salt stress was simulated by treating the plants with a 100 mM NaCl solution, while 1/2 Hoagland nutrient solution was used as a control (CK) instead of the NaCl solution. The results showed that the two rootstocks responded to salt environments by increasing the proportion of root biomass allocation. According to the stress susceptibility index, ‘9-1-6’ exhibits a lower salt sensitivity index and a higher salt tolerance index. The thickness of the leaf, upper and lower epidermis, palisade tissue, and mesophyll tissue compactness (CTR) of the two rootstocks were significantly decreased, while the thickness of sponge tissue and mesophyll tissue looseness (SR) were significantly increased, and the range of ‘9-1-6’ was smaller than that of *M. baccata*. With an extension of stress time, the accumulation of Na^+^ increased significantly, and the accumulation of K^+^ decreased gradually. The stem and leaves of ‘9-1-6’ showed a lower accumulation of Na^+^ and a higher accumulation of K^+^, and the roots displayed a higher ability to reject Na^+^, as well as young and old leaves showed a stronger ability to secrete Na^+^. In conclusion, within a certain salt concentration range, the ‘9-1-6’ root part can maintain lower salt sensitivity and a higher root-to-shoot ratio by increasing the proportion of root biomass allocation; the aerial part responds to salt stress through thicker leaves and a complete double-layer fence structure; the roots and stem bases can effectively reduce the transportation of Na^+^ to the aerial parts, as well as effectively secrete Na^+^ from the aerial parts through young and old leaves, thereby maintaining a higher K^+^/Na^+^ ratio in the aerial parts, showing a strong salt tolerance.

## 1. Introduction

Soil salinization is a significant factor that hinders agricultural development. Approximately 20% of arable land and nearly half of irrigated land worldwide are at risk of salt damage and continued deterioration [1]. According to FAO sources (http://www.fao.org/soils-portal (accessed on 29 March 2024)), in China alone, more than one million acres of agricultural land are affected by salt damage due to irrigation water containing highly soluble salts [2]. In apple production regions, the excessive use of chemical fertilizers and lack of organic fertilizers has led to the spreading problem of secondary soil salinization [3]. This imbalance in nutrition for fruit trees and the decline in fruit quality significantly restrict the further development of the apple industry [4]. Salt tolerance in rootstocks is the cornerstone for fruit trees to adapt to saline–alkali land. Different rootstocks exhibit varying abilities to absorb water and mineral nutrients under salt stress, indirectly impact the sink-source relationship of plants, and affect plant growth, fruit yield, and quality [5,6]. Therefore, it is crucial to study rootstock resources with high resistance to salty soils for the advancement of the apple industry.

Plants usually regulate biomass allocation to adapt to environmental changes, and the root cap ratio is an important indicator for measuring the impact of stress on biomass allocation [7]. In general, plant biomass decreases under salt stress, but the degree of decrease depends on plant genotype, salt concentration, etc. Research shows that under 50 and 150 mmol/L NaCl stresses, the aboveground dry weight of *Quercus virginiana* is significantly higher than that of *Quercus acutissima* [8]. On the other hand, when plants are exposed to salt stress, they typically display a range of adaptive traits in the morphological structure of their roots, stems, leaves, and so on. Leaves, as a highly flexible organ in plant growth and development, the changes in their morphological structure, particularly anatomical features, are closely linked to the salt tolerance of plants [9]. Studies have indicated that under salt stress, the thickness of palisade tissue in the leaves of *Betula microphylla* var. *paludosa* increases [10]. Additionally, the thickness of leaf epidermis, palisade tissue, and sponge tissue in *Vitis vinifera* significantly increases, while the ratio of palisade tissue to sponge tissue significantly decreases [11]. This suggests that conducting research on the anatomical structure characteristics of leaves under salt stress can further elucidate the morphological response and adaptation mechanisms of plants to saline environments.

Under salt stress, Na^+^ ions directly interfere with or competitively inhibit the absorption and transport of nutrient ions such as K^+^, Ca^2+^, and Mg^2+^ by the cell membrane, causing ion imbalance, ion toxicity, and nutrient deficiency in the plant body, thereby affecting the normal growth and development of plants [12,13,14]. Ion toxicity is a primary stress that can lead to secondary stresses such as osmotic stress and nutrient imbalance, mainly manifested as serious harm of Na^+^ and Cl^−^ to plants [15]. However, most plants are non-halophytes, which means their tolerance to salt is relatively low, or their growth is inhibited under reduced salinity [16]. To avoid and cope with salt stress, plants have evolved a series of mechanisms to survive, such as salt dilution, salt excretion, and salt exclusion [17]. Research has shown that the transport and distribution of salt-related ions such as Na^+^, Cl^−^, and K^+^ have their own characteristics at the plant organ and whole plant levels [18,19,20]. There are differences in the response and adaptation of ion metabolism characteristics to salt stress among different varieties or sources of plants, and the main accumulation sites of sodium ions are different [21]. Wang et al. found that the absorption of Na^+^ by tomatoes increased with increases in salt treatment concentration and time [22]. The order of accumulation in each organ was root > stem > leaf, and the total accumulation of Na^+^ in more salt-tolerant varieties was lower than in salt-sensitive varieties. Salt-tolerant fig cultivars intercepted more Na^+^ in the roots, with most of the transported Na^+^ accumulating in the stem base, while the Na^+^ content in the stem apex was very low [23]. Much attention has been paid to sodium accumulation in olives. Under salt stress, all cultivars showed a significant reduction in growth, but to varying degrees. “Lefkolia Serron” exhibited the fewest symptoms, with the lowest sodium concentration in the leaves and the highest in the roots [24].

As an important economic fruit tree, apples (*Malus domestica*) have strong adaptability to various soils. Using salt-tolerant rootstocks for grafting cultivation can effectively reduce the harm caused by soil salinization and fully utilize soil resources. In the apple industry, rootstocks with excellent tolerance have been widely applied to improve plant resistance [25]. The salt tolerance of some apple rootstocks has been previously evaluated, showing large differences among species. However, we have found that some excellent rootstocks have not yet been developed and utilized, and their salt tolerance mechanisms are also unclear. *Malus halliana* Koehne, originating from the Gansu Hexi Corridor in China, is an excellent apple rootstock with strong drought, salt, and cold resistance and is an important plant resource for breeding purposes [26]. *Malus Baccata* Borkh, native to Northeast China, is a common rootstock for apple production. It is cold and drought-tolerant but not salt-tolerant. Our study found that *M. halliana* has higher drought resistance and iron deficiency than other apple rootstocks and grows well in saline–alkali soil [27,28,29]. Currently, research on apples under salt stress mainly focuses on the evaluation of resistance and physiological and ecological characteristics among different varieties [30,31,32]. However, there are few reports on the absorption and accumulation process of related ions in different organs and the salt rejection and secretion characteristics.

Two rootstock varieties, *Malus halliana* ‘9-1-6’ (with strong salt tolerance) and *Malus baccata* (with weak salt tolerance), were used as experimental materials in this study. By studying the growth and Na^+^ and K^+^ absorption and transportation dynamics of rootstock plants under NaCl stress, the absorption and accumulation patterns of Na^+^ and K^+^ in various tissues of apple rootstock under a salt environment, and the salt tolerance mechanism of rootstock were clarified, in order to provide a theoretical basis for breeding and promoting new apple rootstock varieties.

## 2. Results

### 2.1. Effects of Salt Stress on the Salt Injury Index of M. halliana ‘9-1-6’ and M. baccata

**Figure 1:**
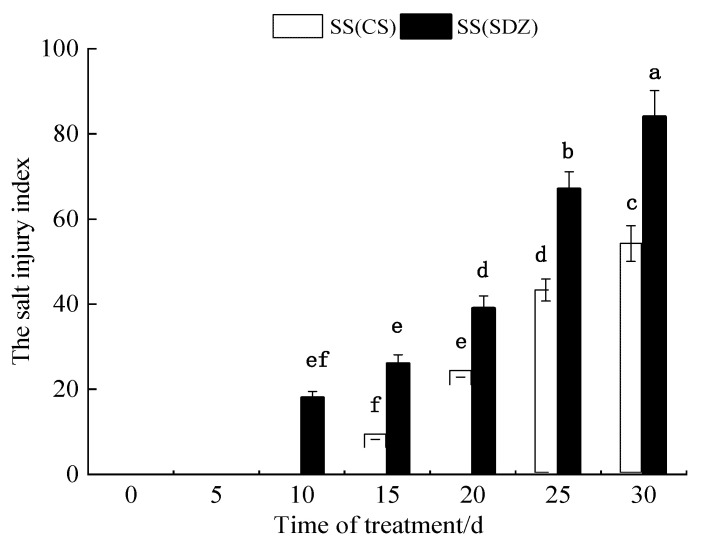
Dynamic changes in salt damage index of two apple rootstocks under NaCl stress. Note: The values are the means ± standard errors, *n* = 3. Different lowercase letters indicate significant differences at the 0.05 level (*p* < 0.05). SS (CS): salt stress *(M. halliana* ‘9-1-6’); SS (SDZ): salt stress *(M. baccata*), d: days.

As can be seen from Figure 1, the salt injury symptoms of *M. baccata* appeared on the 10th day after the 100 mmol·L^−1^ NaCl stress treatment, while the salt injury symptoms of ‘9-1-6’ appeared on the 15th day. With an extension in NaCl stress time, the salt injury indices of the two varieties gradually increased. After 30 days of NaCl stress treatment, the salt injury index of *M. baccata* reached 84.1, and the plants were almost all dead, while that of ‘9-1-6’ was only 54.5. The results showed that the salt tolerance of ‘9-1-6’ was significantly higher than that of *M. baccata* under 100 mmol·L^−1^ NaCl stress (*p* < 0.05).

### 2.2. Effects of Salt Stress on the Growth, Sensitivity, and Tolerance of M. halliana ‘9-1-6’ and M. baccata

As seen in Table 1, after 30 days of treatment with 100 mmol · L^−1^ NaCl stress, the aerial and root dry weight of both varieties decreased to varying degrees, both significantly lower than the control. The aerial dry weight of ‘9-1-6’ is 41.03% of its control, and *M. baccata* is 24.07% of its control. In addition, ‘9-1-6’ is 1.27 times higher than *M. baccata*. The root dry weight of ‘9-1-6’ is 62.21% of its control, while *M. baccata* is 49.73%, which is 0.8 times that of ‘9-1-6’. The root-to-shoot ratios of ‘9-1-6’ and *M. baccata* were significantly higher than that of the control after stress at 1.54 times and 2.12 times, respectively.

From Table 2, after salt stress, the aerial sensitivity index of the two rootstocks is significantly higher than that of the root part. The salt sensitivity index of *M. halliana* ‘9-1-6’ aerial and root parts is notably lower than that of *M. baccata*, at 77.63% and 76.00%, respectively. Additionally, the tolerance indices of the root parts of the two rootstocks were significantly higher than those of the aerial parts. The tolerance indices of the aerial and root parts of *M. halliana* ‘9-1-6’ rootstocks were significantly higher than those of *M. baccata*, at 1.67 and 1.27 times, respectively.

### 2.3. Effect of Salt Stress on the Anatomical Structure of Leaves in M. halliana ‘9-1-6’ and M. baccata

#### 2.3.1. Leaf Epidermal Characteristics

As seen in Table 3, after 30 days of stress, the leaf thickness and upper and lower epidermal thickness of the two rootstocks significantly decreased. Compared with the control, the leaf thickness of ‘9-1-6’ and *M. baccata* decreased by 17.25% and 29.99%, respectively, and the leaf thickness of ‘9-1-6’ was significantly greater than that of *M. baccata*, which was 1.24 times its thickness. The thickness of the upper epidermis of ‘9-1-6’ leaves was significantly greater than that of *M. baccata* before and after stress, 1.47 and 1.52 times, respectively. The thickness of the lower epidermis of both varieties decreased significantly before and after stress, but there was no significant difference between the varieties (*p* < 0.05).

#### 2.3.2. Leaf Mesophyll Characteristics

Under salt stress, the thickness of the palisade tissue and CTR of two rootstock leaves continuously decreased, while the thickness of the spongy tissue and SR continuously increased, with different varieties showing different degrees of change. After 30 days of stress, the palisade tissue cells of ‘9-1-6’ leaves shortened and became densely arranged, while the spongy tissue cells reduced in size and the cell interspace decreased. The palisade tissue cells of *M. baccata* leaves also shortened, with a loose and irregular arrangement, while the spongy tissue cells reduced in size and the cell interspace increased compared with 0 days. Compared with the control, the thickness of the palisade tissue and CTR in the two rootstocks decreased by 29.91% and 50.61%, and 14.00% and 25.40%, respectively, after 30 days of stress. The reduction in thickness and CTR in ‘9-1-6’ leaves were smaller than that of the *M. baccata*, and after the decrease, the palisade tissue thickness and CTR in ‘9-1-6’ leaves were significantly greater than those of the *M. baccata*, at 1.66 and 1.30 times respectively. The thickness of the spongy tissue and SR of the two rootstocks increased significantly by 5.34% and 13.32%, and 37.69% and 58.48%, respectively. The increase in *M. baccata* leaves was higher than that of ‘9-1-6’, and the thickness of the spongy tissue and SR of *M. baccata* leaves were significantly higher than those of ‘9-1-6’, at 1.08 and 1.23 times respectively (*p* < 0.05).

### 2.4. Effects of Salt Stress on Na^+^ Contents in Different Parts of M. halliana ‘9-1-6’ and M. baccata

**Figure 2:**
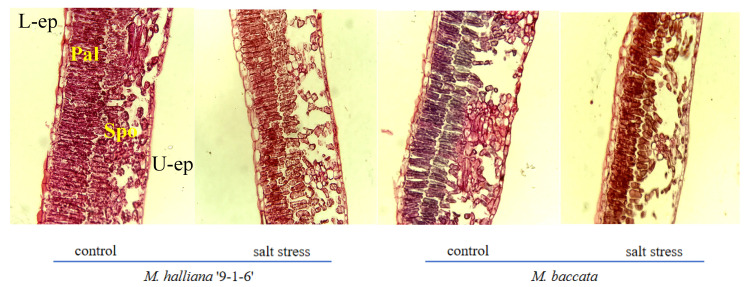
Effect of salt stress on the anatomical structure of leaves of two apple rootstock types (×10). L-ep: Lower epidermis; U-ep: Upper epidermis; Pal: Palisade tissue; Spo: Spongy tissue.

**Figure 3:**
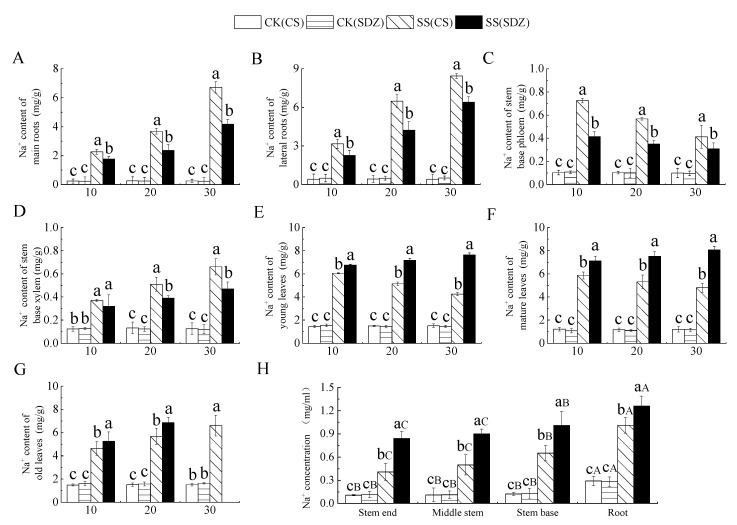
Effect of salinity on Na^+^ content in different parts of two apple rootstocks. (**A**) Na^+^ content of main roots; (**B**) Na^+^ content of lateral roots; (**C**) Na^+^ content of stem base phloem; (**D**) Na^+^ content of stem base xylem; (**E**) Na^+^ content of young leaves; (**F**) Na^+^ content of mature leaves; (**G**) Na^+^ content of old leaves; (**H**) Na^+^ concentration in different parts. Note: The values are the means ± standard errors, *n* = 3. Different lowercase letters indicate significant differences at the 0.05 level (*p* < 0.05) at the same time point (same location, (**H**)). Different capital letters indicate significant differences in different parts (*p* < 0.05). CK (CS): control (*M. halliana* ‘9-1-6’); CK (SDZ): control (*M. baccata*); SS (CS): salt stress (*M. halliana* ‘9-1-6’); SS (SDZ): salt stress (*M. baccata*).

It can be seen from Figure 2 that with the continuous stress, Na^+^ content in different parts of different rootstocks showed different trends. Figure 3A,B showed that the Na^+^ content in the main root and lateral root of the two rootstocks showed an increasing trend, and the Na^+^ content accumulated in the lateral root was higher than that in the main root.

After 30 days of stress, the Na^+^ content in the main root and lateral root was significantly higher than that in CK. The Na^+^ content in the main root and lateral root of ‘9-1-6’ was also significantly higher than that of *M. baccata*, being 1.61 and 1.32 times higher than that of *M. baccata*, respectively. This indicates that the Na^+^ accumulation ability of the ‘9-1-6’ main root and lateral root was significantly higher than that of *M. baccata*. Figure 3C showed that the Na^+^ content in the phloem of the stem base of the two rootstocks reached its peak after 10 days of stress and then decreased continuously. After 30 days of stress, the Na^+^ content in the two rootstocks was significantly higher than their respective CKs, being 4.13 and 3.19 times their respective CKs. ‘9-1-6’ still maintained a higher Na^+^ content level, which was 1.34 times that of *M. baccata*. After 30 days of stress, the Na^+^ content in the xylem of ‘9-1-6’ was 1.40 and 5.24 times higher than that of *M. baccata* and the CK, respectively (Figure 3D).

It is evident from Figure 3E–G that the Na^+^ content in young leaves and mature leaves of ‘9-1-6’ initially increased and then decreased under salt stress, while that in ‘9-1-6’ continued to increase, and both rootstocks showed a significant increase in the first 10 days followed by stabilization. After 30 days of stress, the Na^+^ content in young and mature leaves of both rootstocks was significantly higher than that in CK, and the Na^+^ content in young and mature ‘9-1-6’ leaves was significantly lower than that in *M. baccata*, with reductions of 55.7% and 59.4%, respectively. The Na^+^ content in old ‘9-1-6’ leaves was significantly higher than that in *M. baccata,* its CK after 20 days of stress. After 30 days of stress, the accumulation of Na^+^ in ‘9-1-6’ leaves was higher than in the young and mature leaves, and the old leaves fell off. Figure 3H illustrates the Na^+^ concentration in the xylem sap of different parts after 10 days of salt stress. It is evident that there was no significant difference in Na^+^ concentration in the same part of the two rootstocks in the CK treatment, but there was a significant difference in different parts, with the root showing a significantly higher concentration than the stem. After salt stress, the Na^+^ concentration in different parts of the two rootstocks was significantly higher than that of CK, but the increase in different varieties was different. The concentration of Na^+^ in the root, stem base, middle stem, and tip of ‘9-1-6’ was 0.80, 0.64, 0.56, and 0.49 times lower than that of *M. baccata*, respectively. The concentration of Na^+^ in the root, stem base, middle stem, and tip of ‘9-1-6’ was higher than that of the stem, and the concentration of Na^+^ in the stem base was higher than that of the middle stem and tip.

### 2.5. Effects of Salt Stress on K^+^ Contents in Different Parts of M. halliana ‘9-1-6’ and M. baccata

**Figure 4:**
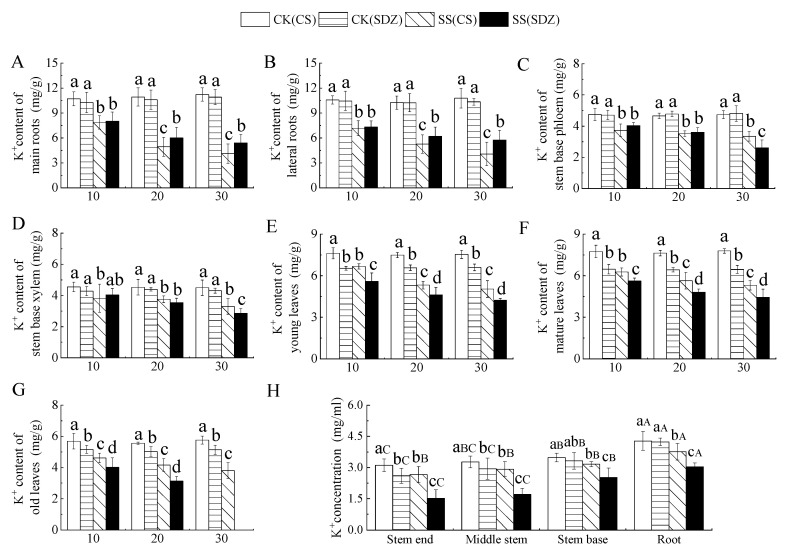
Effect of salinity on K^+^ concentration in different parts of two apple rootstocks. (**A**) K^+^ content of main roots; (**B**) K^+^ content of lateral roots; (**C**) K^+^ content of stem base phloem; (**D**) K^+^ content of stem base xylem; (**E**) K^+^ content of young leaves; (**F**) K^+^ content of mature leaves; (**G**) K^+^ content of old leaves; (**H**) K^+^ concentration in different parts. Note: The values are the means ± standard errors, *n* = 3. Different lowercase letters indicate significant differences (*p* < 0.05) at the same time point (same location, subfigure (**H**)). Different capital letters indicate significant differences in different parts (*p* < 0.05). CK (CS): control (*M. halliana* ‘9-1-6’); CK (SDZ): control (*M. baccata*); SS (CS): salt stress (*M. halliana* ‘9-1-6’); SS (SDZ): salt stress (*M. baccata*).

It is evident from Figure 4 that the K^+^ content in young leaves, mature leaves, and old leaves was significantly lower than that of ‘9-1-6’ in the CK treatment, with no significant difference in K^+^ content in other parts. Under salt stress, K^+^ content in different parts of the two rootstocks showed a decreasing trend, but the decreasing range varied. Figure 4A,B indicate that the K^+^ content in the main root and lateral root of ‘9-1-6’ decreased by 63.28% and 62.27%, respectively, after 30 days of stress, while that of *M. baccata* decreased by 50.33% and 44.37% respectively. After 30 days of stress, the K^+^ content in the main root and lateral root of ‘9-1-6’ was significantly lower than that of *M. baccata* by 76.12% and 70.70%, respectively. Figure 4C,D showed that K^+^ content in the stem base phloem and stem base xylem of the two rootstocks decreased continuously under salt stress. After 30 days of salt stress, K^+^ content in stem base phloem and stem base xylem of ‘9-1-6’ was significantly higher than that of *M. baccata*, by 1.28 and 1.15 times, respectively. Figure 4E,F revealed that K^+^ content in young leaves, mature leaves, and old leaves of the two rootstocks decreased continuously under salt stress. After 30 days of salt stress, the K^+^ content in young leaves and mature leaves of ‘9-1-6’ was significantly higher than that of *M. baccata*, by 1.19 and 1.20 times higher than that of *M. baccata*, respectively. After 30 days of ‘9-1-6’ old leaf stress, it was 65.97% of CK. After 30 days of *M. baccata* old leaf stress, the leaves fell off (Figure 4G).

### 2.6. Effects of Salt Stress on Na^+^/K^+^ ration of M. halliana ‘9-1-6’ and M. baccata

**Figure 5:**
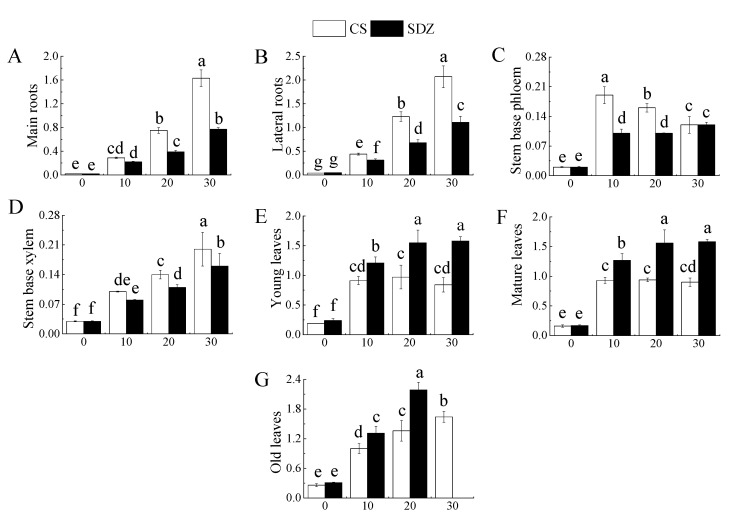
The effect of salt stress on the Na^+^/K^+^ value in different parts of two rootstocks. (**A**) Na^+^/K^+^ ratio of main roots; (**B**) Na^+^/K^+^ ratio of lateral roots; (**C**) Na^+^/K^+^ ratio of stem base phloem; (**D**) Na^+^/K^+^ ratio of stem base xylem; (**E**) Na^+^/K^+^ ratio of young leaves; (**F**) Na^+^/K^+^ ratio of mature leaves; (**G**) Na^+^/K^+^ ratio of old leaves. Note: The values are the means ± standard errors, *n* = 3. Different lowercase letters indicate significant differences at the 0.05 level (*p* < 0.05). CS: *M. halliana* ‘9-1-6’; SDZ: *M. baccata*.

It is evident from Figure 4 that the Na^+^/K^+^ values in different parts of the two rootstocks exhibited varying trends under salt stress. The Na^+^/K^+^ value in the main root and lateral root increased continuously during the stress period. After 30 days of stress, the Na^+^/K^+^ value in ‘9-1-6’ was significantly higher than that of *M. baccata*, being 2.12 and 1.86 times that of *M. baccata*, respectively. The Na^+^/K^+^ value in the lateral root of both rootstocks was higher than that of their main roots, being 1.27 and 1.44 times that of their main roots, respectively (Figure 5A,B).

Throughout the continuous stress, the phloem of the stem base of the two rootstocks displayed different changing trends, with ‘9-1-6’ showing a trend of initially rising and then falling, while the phloem of *M. baccata* showed a rising trend (Figure 5C). The stem base xylem exhibited a continuous upward trend, but the rising ranges of the two varieties were different. Compared with 0 d, the increase was 566.67% and 433.33%, and after rising, ‘9-1-6’ was significantly higher than that of *M. baccata*, being 1.25 times that of *M. baccata* (Figure 5D).

It can be observed from Figure 5E–G that the Na^+^/K^+^ value in young leaves, mature leaves, and old leaves of the two rootstocks increased significantly during the stress process, but the increase amplitude and trend were different. The Na^+^/K^+^ values in the two varieties showed a rising trend. After 30 days of stress, the Na^+^/K^+^ values in the old leaves of ‘9-1-6’ were 1.95 and 1.82 times higher than those of the young leaves and mature leaves, respectively.

The old leaves fell off after 30 days of stress. The Na^+^/K^+^ ratio in young and mature leaves of ‘9-1-6’ increased significantly after 10 days of stress and then did not change significantly, and the *M. baccata* appeared in 20 d. When the stress was 30 d, the Na^+^/K^+^ value in young and mature leaves of *M. baccata* was significantly higher than that of ‘9-1-6′, 1.82 and 1.76 times, respectively.

### 2.7. Difference of Na^+^ Rejection Ability between M. halliana ‘9-1-6’ and M. baccata under Salt Stress

It is evident from Table 4 that the primary Na^+^ rejecting areas of the two rootstocks are the root and the base of the stem. The Na^+^ rejecting capacity of the middle and the end of the stem is notably lower than that of the root and the base of the stem. The root is 2–3 times that of the stem, and the base of the stem is 2–4 times that of the middle or the end of the stem. Under 100 mmol/L NaCl treatment, the Na^+^ rejecting capacity of ‘9-1-6’ reached 82%, which was significantly higher than that of *M. baccata* by 64%. The Na^+^ rejecting capacity of the ‘9-1-6’ root and stem base was significantly higher than that of *M. baccata*, which were 1.56 and 1.45 times higher than that of *M. baccata*, respectively.

### 2.8. Na^+^ Secretion Capacity of Young, Mature, and Old Leaves of the Seedlings of M. halliana ‘9-1-6’ and M. baccata under Salt Stress

**Figure 6:**
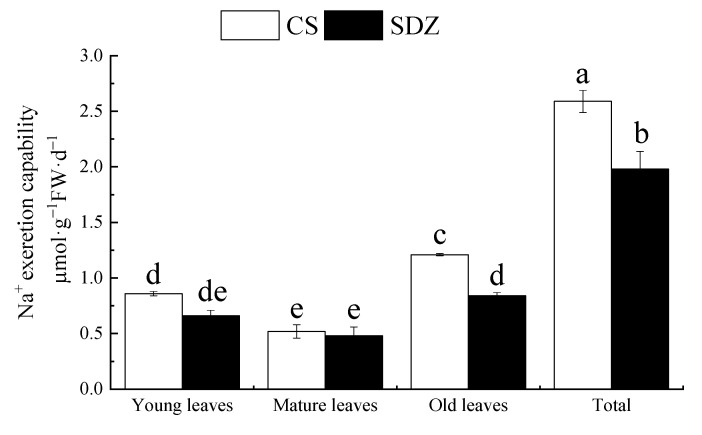
Na^+^ secretion ability of young leaves, mature leaves, and old leaves of two rootstocks under salt stress. Note: The values are the means ± standard errors, *n* = 3. Different lowercase letters indicate significant differences at the 0.05 level (*p* < 0.05). CS: *M. halliana* ‘9-1-6’; SDZ: *M. baccata*.

It can be observed from Figure 6 that there is a significant difference in the Na^+^ secreting capacity of the leaves of the two rootstocks at different maturities, and the total Na^+^ secreting capacity of ‘9-1-6’ is 1.31 times higher than that of *M. baccata*. After 10 days of stress, the Na^+^ secreting capacity of young and old ‘9-1-6’ leaves was higher than that of *M. baccata*, and that of ‘9-1-6’ was 1.44 times higher than that of *M. baccata*. There was no significant difference in mature leaves between the two rootstocks, which were 42.98% and 57.14% of the old leaves and 60.47% and 72.73% of the young leaves, respectively. Under the same level of salt stress, the Na^+^-secreting capacity of the two rootstocks was as follows: old leaves > young leaves > mature leaves.

## 3. Discussion

### 3.1. Effects of Salt Stress on the Growth, SSI, and STI of M. halliana ‘9-1-6’ and M. baccata

Under salt stress, plants can maintain their survival under adverse conditions by slowing down growth and rebuilding biomass allocation [33] and exhibit different biomass allocation patterns, SSI and STI [34]. Under high salt conditions, the proportion of aerial biomass allocation in some plants increases while the proportion of root biomass decreases [35]. However, the biomass allocation pattern of other plants is exactly the opposite and displays an increase in the allocation of biomass to the root system under salt stress [36]. In this study, after 30 days of NaCl stress, the aerial dry weight and root dry weight of two apple rootstock varieties decreased to varying degrees, and the aerial biomass and root biomass of ‘9-1-6’ were significantly higher than *M. baccata*, wang et al. also reached a similar conclusion that *Quercus virginiana* with higher resistance has higher aerial biomass and root biomass [8]. Numerous studies have shown that the SSI of plant roots is lower than that of aerial parts, resulting in an increase in the root-to-shoot ratio under salt stress [8,37]. This study has reached a consistent conclusion. Compared with *M. baccata*, ‘9-1-6’ has a lower SSI and a higher STI and root-to-shoot ratio. Therefore, both rootstock biomass allocation strategies may be to increase the allocation of biomass to the root system. The positive significance of this distribution model may be that it enhances the growth ability of plants and dilutes intracellular salt by increasing root access to water and nutrients, which may be one of the factors contributing to the higher resistance of ‘9-1-6’.

### 3.2. Effects of Salt Stress on the Anatomical Structure of M. halliana ‘9-1-6’ and M. baccata

Salt stress can damage plant leaves and accelerate their aging. Plants in saline environments often have special leaf anatomical features, such as thickening of the cuticle layer, succulent leaves, well-developed palisade tissue, and sunken stomata [38]. These anatomical structures more directly reflect the adaptability of plants to salt stress. In this study, under salt stress, the arrangement of the palisade tissue of the leaves of two apple rootstocks became tighter, the spongy tissue became thicker and looser, and the cells of the palisade and spongy tissues became significantly smaller. This was manifested as a gradual decrease in the thickness of the leaves and palisade tissue, a gradual increase in the thickness of the spongy tissue, a gradual decrease in the CTR value, and a gradual increase in the SR value. These findings are consistent with the results of Parida et al. in American mangroves, which may be due to the water deficiency hindering the water metabolism of the leaves under salt stress to restrict the growth of cells and division, thus limiting the growth of the leaves [39]. In addition, there were differences in the thickness of the leaves and the upper epidermis of the two rootstocks, indicating that the leaf thickness and structure are related to the genotype of the plant. Studies have shown that the thicker the leaf, the better their water storage capacity [40], and well-developed palisade tissue in the leaves can prevent cell water evaporation, which is beneficial for improving photosynthetic efficiency [41]. After 30 days of salt stress in this study, the leaf thickness of ‘9-1-6’ was significantly greater than that of *M. baccata*, with a thicker and well-developed double-layer palisade tissue and a higher CTR, which may reduce the water loss of ‘9-1-6’ leaves, allowing them to maintain a higher photosynthetic rate in a salt stress environment, thereby enhancing their adaptability and salt tolerance.

### 3.3. Effects of Salt Stress on Na^+^ Contents in Different Parts of M. halliana ‘9-1-6’ and M. baccata

Salt stress harms plants in two main ways: ion stress effects and osmotic stress, as well as the resulting oxidative stress and nutritional stress. Among them, ion stress is the primary stress, while osmotic stress, oxidative stress, and nutritional stress are secondary stresses caused by salt ions in the soil [42]. Therefore, under salt stress, the characteristics of plant selection and absorption of salt ions, regional distribution, and other characteristics are key to salt tolerance.

The primary cause of ion toxicity in plants is the excessive absorption of Na^+^. In apple rootstocks, Na^+^ accumulates in both the aboveground and root parts under saline conditions [43]. This study identified the characteristics of ion accumulation in response to salt stress in both varieties. The Na^+^ content in the roots, stems, and leaves of both varieties increased as the stress time increased. However, the salt-tolerant variety ‘9-1-6’ had higher Na^+^ accumulation in the roots and stems than the salt-sensitive variety *M. baccata* and the accumulation in the leaves was lower than that of *M. baccata,* which is consistent with previous studies on ion accumulation in plants under salt stress [43].

In response to salt stress, salt-tolerant plants can limit the root absorption of ions through various levels of membrane systems or restrict the transport and distribution of absorbed ions in the above-ground organs, thereby forming salt-avoidance or salt-resistance mechanisms suitable for their own characteristics [22]. *Festuca arundinacea* Schreb. adapts to salt stress by preferentially absorbing and accumulating a large amount of ions in the stems and leaves. The net accumulation of Na^+^ or total inorganic ions in the stems and leaves is higher than that in the roots, which is beneficial for increasing the osmotic potential difference between the shoots and roots, promoting water transport from the roots to the shoots, improving the water status of the shoots, and promoting growth [44]. After NaCl stress on two pistachio varieties, it was found that the increase in Na^+^ content in the roots and stems of salt-tolerant varieties was greater than that of salt-sensitive varieties [45].

Some plants, such as *Nitraria tangutorum* [46], Nitraria sibirica [47], and Phragmites australis, mainly accumulate Na^+^ in their leaves, while *Quercus dentata* [48] mainly concentrates Na^+^ in their roots. Studies have also indicated that salt-tolerant and salt-sensitive plants have different ion compartmentalization methods, with salt-tolerant plants mainly concentrating Na^+^ in the roots and salt-sensitive plants mainly concentrating Na^+^ in the leaves [49]. In this study, the Na^+^ accumulation of two rootstocks with different resistance levels varied. After 30 days of stress, the order of Na^+^ accumulation in each part of ‘9-1-6’ was root > leaf > stem, while that of *M. baccata* was leaf > root > stem, which is consistent with the research conclusions of Gucci and Tattini on the salt tolerance of olives [50]. After absorbing salt, most of the ‘9-1-6’ root system stores it in the roots and stems, which is a typical salt-repellent type, reducing the damage of salt to the leaves and maintaining normal physiological metabolism.

### 3.4. Effects of Salt Stress on K^+^ Contents in Different Parts of M. halliana ‘9-1-6’ and M. baccata

Excessive absorption of Na^+^ under salt stress inhibits the absorption of K^+^, which is an essential ion in plants [51]. K^+^ plays an important role in physiological processes such as ion balance regulation, cell turgor, and osmoregulation, and it can regulate the salt tolerance of plants [52].

Different genotypes react differently under salt stress. In the Edkany variety, the K^+^ concentration remains unchanged, while in the wild Panali tomato, it slightly increases [53]. Guerrier et al. showed that tomato varieties with strong salt tolerance can absorb more K^+^ from the matrix and maintain a high K^+^/Na^+^ ratio [53]. Wang et al. found in their study on the salt tolerance mechanism of pomegranates and peaches that a doubling of K^+^ is beneficial to improving the balance between Na^+^ and K^+^ in plants and increasing their salt tolerance [54]. It is evident that the rules for ion absorption by plants in response to salt stress can vary based on plant genotype, stress intensity, and stress stage.

In this research, the K^+^ content in different parts of both varieties decreased significantly under salt stress. The decrease in K^+^ content in the root system of ‘9-1-6’ was greater than that of *M. baccata*, while the decrease in K^+^ content in the stem and leaves was less than that of *M. baccata*, consistent with the findings of Peng et al. in maize [51]. This could be attributed to the selective transport of more K^+^ from the roots to the stems and leaves of ‘9-1-6’ while simultaneously inhibiting the transport of Na^+^ to the leaves, maintaining a higher K^+^/Na^+^ ratio in the upper part of the plant, thereby enhancing its tolerance to high-salt environments. After 30 days of stress, the K^+^ accumulation in various parts of ‘9-1-6’ showed a leaf > root > stem pattern, while that of *M. baccata* showed a root > leaf > stem pattern. This may be due to selective absorption, transportation, and regionalization of ions within the plant, with more K^+^ distribution and accumulation in the leaves of salt-tolerant varieties than in sensitive varieties, consistent with the results of Wang et al. in tomato [22].

### 3.5. Effects of Salt Stress on Na^+^/K^+^ Ratio in Different Parts of M. halliana ‘9-1-6’ and M. baccata

Maintaining a normal Na^+^/K^+^ ratio in plants under salt stress is an important indicator of plant salt tolerance. However, due to the similar ionic radii and hydration energies of the two ions, Na^+^ exhibits a significant competitive inhibitory effect on the absorption and active sites of K^+^ under salt stress, resulting in the replacement of K^+^ in root cells by Na^+^, ultimately leading to a decrease in K^+^ absorption and inhibition of enzyme activity and metabolic processes dependent on K^+^ [55]. Therefore, plants on salinized soils are often subjected to the dual harm of Na^+^ toxicity and K^+^ deficiency. Maintaining a high level of K^+^ and K^+^/Na^+^ ratio in cells can reduce the toxicity caused by salt ions, which is necessary for normal physiological metabolism in the body [56].

In this study, as salt stress continued, the Na^+^/K^+^ values in all parts of the two rootstocks significantly increased. Compared with the salt-sensitive rootstock *M. baccata*, the increase in Na^+^/K^+^ values in the roots of ‘9-1-6’ was significantly greater, while the increase in young and mature leaves was less than that of *M. baccata*. This may be due to the stronger selective absorption of K^+^ during the transport of ‘9-1-6’ from the roots to the aboveground parts, which is important for maintaining the balance of nutrients in the body and promoting photosynthesis and growth of leaves. This is consistent with the ion metabolic characteristics of *Hordeum vulgare* [57] and *Corylus heterophylla* × *Corylus avellana* [58] under salt stress. During this process of change, the Na^+^/K^+^ value in the old leaves of ‘9-1-6’ significantly increased. After 10 days of stress, there was no significant difference in the Na^+^/K^+^ values in young and mature leaves, which may be due to the ion compartmentalization in ‘9-1-6’, which distributes more Na^+^ transported to the upper part of the plant to the old leaves, thus maintaining a lower Na^+^/K^+^ ratio in the young and mature leaves. This may be the key to the high salt tolerance of ‘9-1-6’.

### 3.6. Effects of Salt Stress on Na^+^ Rejection and Na^+^ Secretion of M. ‘9-1-6’and M. baccata

The characteristic of plant resistance to Na^+^ is to avoid excessive salt entering the body through root ultrafiltration or interruption of transport, while the salt entering the body will accumulate in different parts under the action of ion compartmentalization, and different plants and varieties often exhibit different compartmentalization patterns. Previous studies on the seedlings of *Malus* plants have shown that their main Na^+^ rejection sites are in the roots and stem base [43]. This study also reached a consistent conclusion that the Na^+^ rejection ability of the root was significantly higher than that of the stem base, and the stem base was significantly higher than the middle and top parts of the stem.

The Na^+^ rejection ability of the highly resistant rootstock ‘9-1-6’ was significantly higher than that of the susceptible *M. baccata*, which was similar to the research conclusion of Ma et al. in different salt-tolerant *Fagopyrum esculentum* Moench Varieties [59]. Research has shown that under salt stress, the activity of H^+^-ATPase, proton pumps, and Na^+^/H^+^ antiporters in the plasma membrane and tonoplast microcapsules in apple root systems significantly increases, and the increase in salt-tolerant species is significantly greater than that in salt-sensitive species. The increase in H^+^-ATPase and proton pump activity is beneficial for establishing a transmembrane proton electrochemical gradient, which better provides energy for the Na^+^/H^+^ antiporter in the plasma membrane and tonoplast. High plasma membrane and tonoplast Na^+^/H^+^ antiporter activity is beneficial for Na^+^ efflux and Na^+^ compartmentalization in the root system [43]; this may be the reason why ‘9-1-6’ has a strong salt rejection ability.

Salt secretion is an important mechanism in plant salt tolerance. Studies have shown that salt-secreting plants secrete salt through salt-secreting structures such as salt glands, glandular hairs, and vesicles [60]. In addition, there is also salt secretion through the waxy layer of the leaf epidermis [61], leaf stomata, and leaf gaps [62] to adapt to the high-salt habitat in which they grow. Studies on plants of the genus *Malus* have shown that their leaves have a Na^+^ secretion phenomenon [43], which has also been confirmed in this study. We compared the sodium ion secretion ability in leaves of two different maturity levels of rootstocks and found that the Na^+^ secretion ability was ranked as follows: old leaves (lower leaves) > young leaves > mature leaves. Old leaves secrete more sodium ions, likely due to their higher accumulation of Na^+^, and their stomata, water pores, or cell gaps focus on salt secretion rather than photosynthesis. Young leaves may be active sites of metabolism and have strong transpiration, while mature leaves secrete less, probably to ensure the normal functioning of photosynthetic gas exchange and not to participate in salt secretion [63]. Additionally, under the same level of treatment, the ability of high-resistance line ‘9-1-6’ old leaves to secrete Na^+^ was significantly greater than that of sensitive line *M. baccata*, which may be due to different physiological and morphological responses of different varieties under salt stress and ion compartmentalization.

## 4. Materials and Methods

### 4.1. Plant Materials

One-year-old salt-tolerant species *Malus halliana* ‘9-1-6’ and salt-sensitive species *Malus baccata* (L.) Borkh. were used as experimental materials. Among them, ‘9-1-6’ is a tissue culture rapid propagation seedling of *Malus halliana* in the Wuwei habitat, with strong salt resistance, and was obtained by comparing the salt resistance of *Malus halliana* in different habitats. This experiment was performed in the greenhouse of Gansu Agricultural University (Gansu Province, China) in April 2020. Plants were transplanted in pottery basin pots with a diameter of 25 cm containing 3.5 kg of nutrient matrix (the volume ratio of vermiculite, perlite, and peat is 1:1:3), with a pH value of 7.0. Each pot contained one plant. The pots were well drained with holes at the bottom.

### 4.2. Experimental Design

Stress treatments were conducted on 20 May 2020. Forty plants of uniform height (30 cm) and healthy plants were individually potted; each species had 20 pots and was randomly divided into 4 sets. Two sets were used as control, and the remaining 2 sets were used for salt stress treatments. Each treatment was repeated ten times.

### 4.3. Stress Treatments

This experiment involved two groups: control (CK) and salt stress (SS, provided by NaCl). One level of salinity and the control were utilized in this experiment. Based on previous results, we selected 100 mM to conduct this experiment. The control groups were watered with only 500 mL of Holland, while the stress groups were watered with 500 mL of Holland solution containing the corresponding salts. All groups were treated between 17:00–18:00 every three hours. In the early stage of salt stress, in order to avoid salt shock, the plants were irrigated incrementally at a salt concentration of 25 mM each time until the required salinity concentration (100 mM) was reached, and the stress time was calculated. All parameters were measured after 10, 20, and 30 days of treatment.

### 4.4. Measurement of Indices

#### 4.4.1. Salt Hazard Index Survey

After the salt stress treatment began, the seedlings were investigated every 5 days for salt injury levels. Level 0: no salt injury symptoms; Level 1: mild salt injury, with a small part of the leaf tips, margins, or veins turning yellow; Level 2: moderate salt injury, with about half of the leaf tips and margins scorched; Level 3: severe salt injury, most of the leaf tips and margins scorched or leaves falling off; Level 4: extremely severe salt injury, with branches and leaves scorched and eventually dead.

The salt injury index (SI) = (N1 + N2 × 2 + N3 × 3 + N4 × 4)/(N × 4) × 100.

where N1, N2, N3, and N4 were the numbers of plants that scored 1, 2, 3, and 4, respectively, and N was the total number of plants that were tested for each genotype and treatment.

#### 4.4.2. Measurement of Biomass

After this experiment, three experimental seedlings were randomly selected from each treatment. The roots, new shoots, and leaves of the plants were washed with deionized water, and the surface moisture was absorbed with filter paper. They were then placed in an envelope and numbered in an oven. After killing at 110 °C for 15 min, they were dried at 70 °C to a constant mass (DW) for weighing and recording data, which was used to calculate the biomass of a single plant [64]. The calculation formula is as follows:

Root to shoot ratio = root biomass/(stem biomass + leaf biomass).

#### 4.4.3. Salt Sensitivity Index (SSI) and Salt Tolerance Index (STI) calculation

The Molhtar [31] and Khayat [65] methods were used to calculate the salt sensitivity index (SSI) and salt tolerance index (STI):

SSI = (DW_NaCl_ − DW_control_)/DW_control_

STI = DW_NaCl_/DW_control_

In the formula, DW_NaCl_ represents the dry weight of plants under salt treatment, and DW_control_ represents the dry weight of control plants.

#### 4.4.4. Measurement of Anatomical Structural Parameters of Leaves

In reference to the conventional paraffin section method [66], mature leaves at the 2nd~3rd node from the top were selected and cut into small blocks (0.5 cm × 0.5 cm) along the mid-vein of the leaves with a double-edged blade. The small blocks were then fixed in formalin-acetic acid-alcohol (FAA) fixation solution, vacuumed, and sliced to a thickness of 8~10 µm. Safranin-fast green double staining, sealed with neutral gum and observed under a fluorescence microscope (Revolve RVL-100-G, Echo, San Diego, CA, USA) for anatomical structure photography. The leaf index parameters were measured using a microscopic Vernier caliper, and each structural parameter was the average of 20 observation fields.

Leaf tissue structure tightness (CTR)/% = (Palisade tissue thickness/Leaf total thickness) × 100;

Leaf tissue structure looseness (SR)/% = (Spongy tissue thickness/Leaf total thickness) × 100.

#### 4.4.5. Extraction of Xylem Sap

The xylem sap was extracted using the “pressure chamber” method [67]. On the 10th day, after the salt stress reached the final concentration (100 mM), the root, stem base, stem middle, and stem end were cut off, and xylem sap was extracted using a pressure equal to the absolute value of water potential in that part. 30 µL of xylem sap was collected and diluted to 3 mL with ultrapure water. The Na^+^ concentration was measured using an atomic absorption spectrometer (Jenna Zenith 700P, Jena, Germany).

#### 4.4.6. Determination of Na^+^, K^+^ Content and Na^+^/K^+^ Calculation

At 10d, 20d, and 30d after stress, the main root, lateral root, stem base phloem, stem base xylem, young leaves (leaves growing and developing at the top), mature leaves (leaves formed in the middle), and old leaves (leaves with declining function at the bottom) were taken, respectively. Wash quickly with deionized water and absorb surface moisture with absorbent paper. After 10 min of blanching in a 100 °C oven, bake at 70 °C to a constant weight. 0.1 g of the sample was taken, and the wet digestion method was used to digest it; then, it brought a constant volume of 50 mL, which stood for 3–5 min before the supernatant was collected. An atomic absorption spectrometer was used to measure the Na^+^ and K^+^ contents and calculate the Na^+^/K^+^ values in different tissues from the two rootstocks after 10, 20, and 30 days of stress.

#### 4.4.7. Calculation of Na^+^ Rejecting Ability of Roots and Various Parts of the Ground and Overall Na Rejecting Ability

The root’s ability to reject Na^+^ is expressed as the percentage of the difference between the external Na^+^ concentration and the Na^+^ concentration in the root xylem sap, divided by the external Na^+^ concentration [43].

Na^+^ resistance ability of root = {(1 − [Na^+^]_Wood liquid_/[Na^+^]_External fluid_) × 100%}

The Na^+^ rejecting ability of the stem base was calculated by subtracting the Na^+^ rejecting ability of the root from the Na^+^ concentration of the xylem sap of the stem base, and the Na^+^ rejecting ability of the middle and end of the stem was calculated in turn.

#### 4.4.8. Calculation of Total Na^+^ Excretion Capacity

On the day when the final concentration of NaCl was reached, the salt on the surface of the leaves was washed with ultrapure water and marked. After 10 days of treatment, the labeled young, mature, and old leaves were taken and quickly weighed, and then 100 mL of redistilled water was used to wash the salt and determine the content of Na^+^ in the leaves and the redistilled water [43].

Total Na^+^ secreting capacity = Na^+^ secreting capacity of the shoot (young leaf + mature leaf + old leaf) (µ mol·g^−1^ FW·d^−1^).

### 4.5. Statistical Analysis

Data were analyzed using the statistical software SPSS 22.0 (SPSS, Chicago, IL, USA). Differences between means of treatments were performed by Duncan’s multiple tests at *p* < 0.05. Charts were made using Origin 8.0 software.

## 5. Conclusions

Under salt stress, compared with *M. baccata*, ‘9-1-6’ maintained a higher root-to-shoot ratio through higher root biomass allocation, and it can adapt to salt environments by maintaining a more complete double-layered fence tissue and higher CTR and leaf thickness. The strong sodium ion exclusion ability at the root and stem base of ‘9-1-6’, as well as the strong sodium ion secretion in young and old leaves, maintains a relatively high potassium-sodium ion ratio in the aboveground parts of plants, demonstrating higher salt tolerance.

## Figures and Tables

**Table 1:** Effects of NaCl stress on the growth of *M. halliana* ‘9-1-6’ and *M. baccata*.

NaCl Concentration (mMol/L)		Aerial Dry Weight/g	Root Dry Weight/g	Root/Aerial
*M. halliana* ‘9-1-6’	0 (CK)	35.61 ± 1.32 a	9.42 ± 0.71 a	0.26 ± 0.01 c
	100 (SS)	14.61 ± 2.02 b	5.86 ± 0.34 b	0.40 ± 0.02 b
*M. baccata*	0 (CK)	35.39 ± 0.21 a	9.37 ± 0.31 a	0.26 ± 0.01 c
	100 (SS)	8.52 ± 0.46 c	4.66 ± 1.21 c	0.55 ± 0.04 a

Note: The values are the means ± standard errors, *n* = 3. Different lowercase letters indicate significant differences within the same column (*p* < 0.05). CK: Control, SS: Salt stress.

**Table 2:** Effects of NaCl stress on sensitivity and tolerance of *M. halliana* ‘9-1-6’ and *M. baccata*.

NaCl Concentration (mMol/L)	*M. halliana* ‘9-1-6’		*M. baccata*	
	Aerial Part	Root Part	Aerial Part	Root Part
Salt Sensitivity Index (SSI)	0.59 ± 0.04 b	0.38 ± 0.01 c	0.76 ± 0.08 a	0.50 ± 0.01 b
Salt Tolerance Index (STI)	0.41 ± 0.01 b	0.62 ± 0.03 a	0.24 ± 0.01 c	0.49 ± 0.02 b

Note: The values are the means ± standard errors, *n* = 3. Different lowercase letters on the same line indicate significant differences (*p* < 0.05).

**Table 3:** Effects of salt stress on leaf epidermis and mesophyll thickness of two apple rootstocks.

Anatomical Structure of Leaves	Genotype	Treatment	
		Control	Salt Stress
Thickness of Leaf/µm	*M. halliana* ‘9-1-6’	225.16 ± 4.31 a	186.31 ± 3.24 c
	*M. baccata*	215.12 ± 4.50 b	150.61 ± 3.11 d
Thickness of Upper Epidermis/µm	*M. halliana* ‘9-1-6’	17.11 ± 1.80 a	11.72 ± 1.01 b
	*M. baccata*	11.51 ± 1.09 b	7.69 ± 0.89 c
Thickness of Lower Epidermis/µm	*M. halliana* ‘9-1-6’	8.75 ± 1.14 a	7.07 ± 1.71 b
	*M. baccata*	8.52 ± 1.17 a	6.57 ± 1.02 b
Thickness of palisade/µm	*M. halliana* ‘9-1-6’	128.84 ± 3.98 a	90.31 ± 2.78 c
	*M. baccata*	110.42 ± 3.03 b	54.54 ± 2.64 d
Thickness of spongy/µm	*M. halliana* ‘9-1-6’	64.56 ± 1.86 a	68.01 ± 1.54 ab
	*M. baccata*	64.87 ± 2.01 a	73.51 ± 2.01 b
Cell tightness rate, CTR/%	*M. halliana* ‘9-1-6’	58.41 ± 1.85 a	50.23 ± 1.31 b
	*M. baccata*	51.86 ± 1.42 ab	38.69 ± 2.35 c
Scattered rate, SR/%	*M. halliana* ‘9-1-6’	29.13 ± 1.62 c	40.11 ± 1.09 b
	*M. baccata*	31.09 ± 1.22 c	49.27 ± 2.21 a

Note: The values are the means ± standard errors, *n* = 3. Different lowercase letters indicate significant differences at the 0.05 level (*p* < 0.05).

**Table 4:** Ability to reject Na^+^ in different parts of two rootstocks.

Rootstocks	NaCl Treatment (mmol/L)	Na^+^-Exclusion Capability (%)				
		Roots	Basal Part of Stem	Central Stem	Stem Apex	Total
*M. halliana* ‘9-1-6’	100	0.56 ± 0.11 a	0.16 ± 0.03 c	0.06 ± 0.01 e	0.04 ± 0.002 e	0.82
*M. baccata*	100	0.45 ± 0.08 b	0.11 ± 0.01 d	0.05 ± 0.01 e	0.03 ± 0.001 e	0.64

Note: The values are the means ± standard errors, *n* = 3. Different lowercase letters indicate significant differences at the 0.05 level (*p* < 0.05).

## Data Availability

Data are contained within the article.
